# Inhibition of human retinal pigment epithelial cell attachment, spreading, and migration by the human lectin galectin-1

**Published:** 2009-10-23

**Authors:** Claudia S. Alge-Priglinger, Sabine André, Thomas C. Kreutzer, Cornelia A. Deeg, Anselm Kampik, Marcus Kernt, Harald Schöffl, Siegfried G. Priglinger, Hans-Joachim Gabius

**Affiliations:** 1 Department of Ophthalmology, Ludwig-Maximilians-University, Munich, Germany; 2 Department of Ophthalmology, General Hospital Linz, Linz, Austria; 3 Institute of Physiological Chemistry, Faculty of Veterinary Medicine, Ludwig-Maximilians-University, Munich, Germany; 4 Institute of Animal Physiology, Faculty of Veterinary Medicine, Ludwig-Maximilians-University, Munich, Germany; 5 BioMed-zet-Life-Science-Laboratory, Linz, Austria

## Abstract

**Purpose:**

Adhesion and migration of dislocated retinal pigment epithelial (RPE) cells are initial steps in the pathogenesis of proliferative vitreoretinopathy (PVR). The role of the endogenous lectin, galectin-1, in attachment, spreading, and migration of human RPE cells was investigated from a therapeutic perspective.

**Methods:**

Human RPE cells were treated with galectin-1 concentrations that ranged 0–250 µg/ml. Cell viability was tested by 3-[4,5-dimethylthiazol-2-yl]-2,5-diphenyltetrazoliumbromide (MTT) assay. Galectin-1 binding to the RPE cells was investigated by immunocytochemistry. Attachment of RPE cells was assessed on 96-well plates coated with laminin, or fibronectin, or galectin-1, or the glycoprotein-lectin combinations and subsequent MTT-testing. RPE migration in the absence or presence of galectin-1 on the respective substrates was tested using a modified Boyden chamber assay with platelet derived growth factor (PDGF)-BB as the chemoattractant. Cellular spreading was characterized by cytoplasmic halo formation of RPE cells after three hours in contact with the surface coating.

**Results:**

Galectin-1 bound to the cell surface. Binding could be inhibited by a β-galactoside. MTT assays revealed no toxicity within control limits for the concentration range tested. When added to the medium, galectin-1 dose-dependently inhibited RPE cell attachment, spreading, and migration by more than 70%, irrespective of the substratum tested. When coated onto the plastic surface, galectin-1 alone impaired spreading and migration of RPE cells, and reduced attachment to and migration on fibronectin by up to 80%.

**Conclusions:**

Galectin-1 inhibits RPE cell attachment, migration, and spreading in vitro with no apparent cytotoxicity. This activity of the endogenous effector deserves consideration as a potential therapeutic agent for the prevention of PVR.

## Introduction

Despite continued advances in surgical techniques, proliferative vitreoretinopathy (PVR) remains a major cause of persistent reduction in visual acuity after retinal detachment or severe ocular trauma [1]. Clinically, PVR is characterized by the dispersion of a variety of cells into the vitreous and subsequent formation of scar-like fibrocellular membranes on the surfaces of the neuroretina, which gives rise to subsequent tractional retinal detachment or re-detachment [2,3].

PVR membranes typically contain a predominance of retinal pigment epithelial (RPE) cells. Thus, it is assumed that the development of PVR critically depends on the capacity of these cells for proliferation, migration, and matrix remodeling [4]. Following a retinal injury, RPE cells, which normally form a monolayer of polarized, highly specialized nonproliferating cells at Bruch’s membrane, disseminate and attach to multiple loci on the neuroretina and the vitreous. There, they undergo a pseudometaplastic transformation into fibroblast-like cells, which then spread along the retinal surface, actively dividing and migrating. These processes are suggested to be the key cellular events in the onset of PVR [2,3,5-7].

Blocking these early cellular responses could be viewed as a potential therapeutic target for improving the overall prognosis of surgical treatment. In the past, pharmacologic prevention of PVR membrane formation has been based chiefly on anti-proliferative and anti-inflammatory agents [8-12]. However, the results from these approaches have been disappointing, largely because of their ineffectiveness or their ensuing retinal toxicity [8,13]. As a consequence, no pharmacological adjuvant has yet been established as part of a routine treatment for PVR, and a need remains for nontoxic agents that will specifically block cellular activities such as RPE proliferation and attachment in PVR. One promising approach would involve the use of endogenous effectors that normally exert control over cell movement and proliferation. Our report focuses a protein that is known to regulate cell adhesion, attachment, migration, and proliferation.

Accumulating evidence reveals the role of cell surface glycans as versatile biochemical signals that operate via translation of sugar-encoded information by carbohydrate-binding proteins (lectins) [14]. The range of lectin-protein recognition processes in situ covers an extensive activity profile [14,15]. In this respect, the galectin family figures prominently, due to their multifunctionality. These proteins are characterized functionally by their affinity for β-galactosides and structurally by their β-sandwich fold with a central Trp residue [16,17].

Because they are often positioned at branch ends of glycan chains, β-galactosides or derivatives thereof represent ideal docking sites for galectins [18,19]. Of the many members of the galectin family, galectin-1 is perhaps the best characterized, and is found expressed intracellularly, on the cell surface, and also within the extracellular matrix [20,21]. When externalized, galectin-1 participates in cell-cell and cell-matrix interactions through binding with distinct glycoconjugates present on the cell surface as well as in the extracellular matrix (ECM). These glycoconjugates may include neutral glycolipids and ganglioside GM1 [22,23], the α1/α5/α7/β1-integrin subunits in a cell-type specific manner [24], laminin (Lam) [25], fibronectin (Fn) [26], or suitably glycosylated mucin or chondroitin sulfate proteoglycan chains, as well as several immune cell CD markers. Many of these listed components have been shown to be present in PVR membranes [27].

Depending on the counter-receptor and its glycosylation, galectin-1 can differentially affect a variety of cellular functions, including cell proliferation [24,28,29], adhesion and migration [24,30,31], apoptosis in activated T lymphocytes [21,32], and induction of anoikis in pancreatic carcinoma cells [33]. However, it must be noted that these effects are highly cell type-specific and therefore the response to galectin-1 binding may vary with the cell type under study.

Most of the cell-adhesion molecules and ECM proteins that favor the adhesion and migration of RPE cells contain substantial numbers of β-galactosyl residues, which are ligands for lectins in general and galectins in particular. Previous work with the agglutinin from the edible mushroom *Agaricus bisporus* has revealed substantial inhibition of RPE migration and proliferation [11,34,35]. We have previously demonstrated the presence of galectin-1 in PVR membranes and also have shown that human RPE cells produce galectin-1 both in vivo and in vitro [36]. Galectin-1 has been implicated in the regulation of cell adhesion, migration, and cell growth in a wide variety of cell types, including activated T cells, vascular smooth muscle, neuroblastoma, and pancreatic carcinoma cells [24,29,32,33].

It was therefore of interest to further investigate the potential role of extracellular galectin-1 in the in vitro behavior of RPE cells. Treatment with an endogenous lectin, such as galectin-1, may disclose a new perspective for re-programming key cellular PVR-related events, using an endogenous substance synthesized by RPE cells themselves. Our report is a first step toward establishing the validity of this hypothesis.

## Methods

### Galectin-1 purification, labeling, and quality controls

Galectin-1 was purified after recombinant production by affinity chromatography on lactosylated Sepharose 4B as critical step and elution with 100 mM lactose in PBS (20 mM KH_2_PO_4_/Na_2_HPO_4_, 150 mM NaCl, pH 7.2) followed by dialysis against the same buffer without lactose. The purity of the final product was checked by gel electrophoresis on a 15% polyacrylamide running gel and mass spectrometry on a YTOF instrument type Q-Tof2 (Waters Micromass, Manchester, UK), as described previously [30,37]. Biotinylation was performed under optimized activity-preserving conditions with biotinyl-N-hydroxysuccinimide ester (0.5 mg/mg of protein; Sigma, Deisenhofen, Germany), predissolved in dimethylformamide, in carbonate reaction buffer (0.1 M NaHCO_3_; pH 8; 0.9% NaCl, 20 mM lactose). The degrees of labeling and carbohydrate-binding activity were measured by two-dimensional gel electrophoresis and solid-phase and cell assays, as previously described [38,39]. In detail, two-dimensional gel electrophoresis was performed using an Amersham Biosciences IPGphorTM isoelectric focusing (IEF) unit (Freiburg, Germany) for the first dimension followed by separation on 12.5% polyacrylamide running gels for the second dimension using an Amersham Biosciences Hoefer SEM 600 model (Freiburg, Germany). Gels were stained by application of reagents of a silver staining kit (Amersham Biosciences) and were processed using an ImageScanner (Amersham Biosciences). For solid-phase assays, the surface of microtiter plate wells (Greiner Bio-One, Frickenhausen) was coated with 50 µl PBS (20 mM, pH 7.2) containing the lectin-reactive glycoprotein (0.5µg asialofetuin obtained by desialylation of commercial fetuin; Sigma) at 4 °C overnight. Following blocking residual sites for protein adsorption by an incubation with 100 µl buffer containing 1% (w/v) carbohydrate-free BSA (Sigma) for 1 h at 37 °C and washing, 50 µl of solution containing biotinylated galectin-1 was applied at a concentration resulting in signal intensity within the linear range, specifically bound galectin-1 was detected by its biotin label using streptavidin-peroxidase conjugate (0.5 µg/ml for 1 h at 37 °C; Sigma) and the chromogenic substrates o-phenylenediamine (1 mg/ml; Sigma)/ H_2_O_2_ (1 µl/ml) at 490 nm with a microplate reader (Bio-Rad, Munich, Germany). For cell surface binding, suspensions with 4×10^5^ cells per sample were carefully washed to remove any interfering serum compounds, the extent of protein binding by nonspecific interaction was reduced by an incubation step in Dulbecco's phosphate-buffered saline (9.2 mM Na_2_HPO_4_, 1.5 mM KH_2_PO_4_, 137 mM NaCl, and 2.7 mM KCl at pH 7.4) containing 100 µg/ml of carbohydrate-free BSA, and the biotinylated galectin-1 was incubated with the cells for 30 min at 4 °C to minimize endocytic uptake. Quantitative determination of carbohydrate-dependent galectin-1 binding using streptavidin/R-phycoerythrin (1:40) as a fluorescent marker (Sigma) was performed by flow cytofluorometry in a FACScan instrument (Becton-Dickinson, Heidelberg, Germany).

### Isolation of human RPE cells and human RPE cell culture

Eyes from five human donors (three male, two female) were obtained from the Munich University Hospital Eye Bank and were processed within 4–16 h after death. Donors ranged from 22 to 59 years of age. None of the donors had a known history of eye disease. Methods for securing human tissue were humane, included proper consent and approval, complied with the Declaration of Helsinki, and were approved by the local ethics committee. Human RPE cells were harvested following the procedures described previously [40,41].

In brief, whole eyes were thoroughly cleansed in 0.9% NaCl solution, immersed in 5% poly(1-vinyl-2-pyrrolidone)-iodine (Jodobac, Bode-Chemie, Hamburg, Germany), and rinsed again in the sodium-chloride solution. The anterior segment from each donor eye was removed and the posterior poles were examined with the aid of a binocular stereomicroscope to confirm the absence of gross retinal disease. Next, the neural retinas were carefully peeled away from the RPE-choroid-sclera using fine forceps. The eye cup was rinsed with Ca^2+^ and Mg^2+^ -free Hank’s balanced salt solution, and filled with 0.25% trypsin (GibcoBRL, Karlsruhe, Germany) for 4 min at 37 °C. The trypsin was carefully aspirated and replaced with Dulbecco’s modified Eagles medium (DMEM, Biochrom, Berlin, Germany) supplemented with 20% fetal calf serum (FCS, Biochrom). Using a pipette, the media was gently agitated, releasing the RPE into the media by avoiding damage to Bruch’s membrane. The RPE cell suspension was transferred to a 6-well plate containing 2 ml of DMEM (Biochrom) supplemented with 20% FCS (Biochrom), checked for cross contamination using a microscope and maintained at 37 °C and 5% CO_2_. After reaching confluency, primary RPE cells were subcultured and maintained in DMEM (Biochrom) supplemented with 10% FCS (Biochrom) at 37 °C and 5% CO_2_. Epithelial origin was confirmed by immunohistochemical staining for cytokeratin using a pan-cytokeratin antibody (Sigma). The cells were tested and found free of contaminating macrophages (anti-CD11; Sigma) and endothelial cells (anti-von Willbrand factor, Sigma; data not shown).

Primary RPE cells were subcultured and maintained in Dulbecco’s modified Eagles medium (DMEM; Biochrom, Berlin, Germany) supplemented with 10% fetal calf serum (FCS; Biochrom) at 37 °C and 5% CO_2_. RPE cells of passages 3–7 were used for experiments. When indicated, different concentrations of galectin-1 were included in the culture medium. The tetrazolium dye-reduction assay, 3-[4,5-dimethylthiazol-2-yl]-2,5–126 diphenyltetrazoliumbromide (MTT; Sigma-Aldrich), was used to test for cell viability [36]. For MTT-testing RPE cells were grown on 96 well plates. After the respective incubation times with or without galectin-1 in the medium cells were rinsed with PBS, and 150 µl of MTT working solution (1.5 ml MTT stock, 2 mg/ml in PBS, plus 28.5 ml DMEM) was added and incubation was continued at 37 °C for 20 min. Within this incubation period cells were repeatedly monitored by light microscopy to assure that the blue formazan crystals did not form outside of the cell. The formazan crystals were then dissolved by the addition of dimethyl sulfoxide (DMSO, 70 µl/well). Absorption was measured by a scanning multiwell spectrophotometer at 550 nm (Molecular Probes, Garching, Germany). Results were expressed as the mean percentage of untreated control cells.

### Immunocytochemical galectin-1 localization

RPE cells were seeded on glass coverslips at a density of 2×10^4^ cells per well and grown for 16 h. Cells were washed three times (three minutes each wash) in 0.1% BSA (BSA) and phosphate buffered saline (PBS; 137 mM NaCl; 2.7 mM KCl, 10 mM Na_2_HPO_4_; 2 mM KH_2_PO_4_; pH 7.4) at room temperature. Thereafter cells were treated with 20 μg/ml biotinylated galectin-1 added to DMEM/0.4% FCS for 35 min at 37 °C. After two washes in PBS, nuclei were counterstained with 1 µg/ml Hoechst 33342 (Sigma), followed by a 10 min incubation at room temperature. Following another two washes in PBS (5 min each wash) at RT, cells were fixed in 2% paraformaldehyde, stained with Alexa-Fluor 488™-labeled streptavidin (Invitrogen, Carlsbad, CA) according to the manufacturer’s instructions, and mounted in Kaiser gelatin (Merck, Darmstadt, Germany). Galectin-1-dependent signals were monitored by fluorescence microscopy (Leica, Wetzlar, Germany).

### Cell adhesion assay

Surfaces of 96-well plates (Nunc, Wiesbaden, Germany) were coated with either Fn, Lam (Sigma), or galectin-1 for 16 h at 4 °C. Additionally, Fn- and Lam-coated wells were initially incubated with 0.2 or 2 µg/cm^2^ galectin-1 before the cells were plated. Fn and Lam were solubilized in 1× PBS (pH 7.4) to yield a final density of 2 µg/cm^2^. The plates were air dried, washed with 1× PBS and preincubated with 1% BSA/PBS for one hour before plating the cells. Trypsinized RPE cells were washed once in DMEM with 10% FCS to quench trypsin activity. Suspensions of 2×10^4^ cells were initially incubated in the presence or absence of galectin-1 concentrations, ranging 0–250 µg/ml, or 100 mM β-lactose (the hapten to block carbohydrate-dependent binding of galectin-1) for 30 min at 37 °C and 5% CO_2_. Cells were then added to the wells and allowed to attach for 1 h at 37 °C and 5% CO_2_.

As part of the process to determine cell adhesion, we carefully washed plates two times with PBS using an automated plate washer (Molecular Devices, Ismaning, Germany). The extent of adherence was measured by MTT assay, as described for cell viability testing. The number of attached living cells was proportional to the absorbance of Formazan at 550 nm, as determined by a scanning multiwell spectrophotometer (Molecular Devices). Experiments were performed in triplicate and repeated at least three times.

### Cell migration assay

Migration was assayed by a modification of the Boyden chamber method, using microchemotaxis chambers (Neuro Probe, Gaithersburg, MD) and polycarbonate filters (Nucleopore, Karlsruhe, Germany) with a pore size of 8.0 µm [42]. Before filters were placed between the chambers, they were either left uncoated, or they were coated with either Fn, Lam, galectin-1, or mixtures of glycoprotein and galectin-1 to yield a final density of 2 µg/cm^2^ of each substratum. DMEM containing 20 ng/ml platelet derived growth factor-BB (PDGF-BB, PeproTech, London, UK) was placed in the lower chamber [43], and 5×10^4^ cells, which had been previously incubated with different concentrations of galectin-1 or β-lactose, as described for cell adhesion, were placed into the upper chamber. The chamber was incubated at 37 °C and 5% CO_2_ for 5 h. The filters were then removed, and the RPE cells on the upper side of the filter were scraped off with a cotton-tipped swab. RPE cells that migrated to the lower side of the filter were fixed in situ in methanol and stained with hematoxylin. Five randomly chosen fields were examined for cell density at 200× magnification with a phase-contrast microscope (Leica). Experiments were performed in duplicate and repeated at least four times.

### Cell spreading assay

Cell spreading was assayed on four chamber uncoated slides or on slides coated with either Fn, Lam, or galectin-1 to yield a final concentration of 2 µg/cm^2^. Alternatively, Fn- and Lam-coated wells were initially incubated with 0.2 or 2 µg/cm^2^ galectin-1 before the cells were plated. RPE cells were cultured as described in the human RPE cell culture section. Cells were isolated by trypsinization, which involved 0.25% trypsin in 5 mM EDTA, washed once in DMEM with 10% FCS and twice with medium containing 0.4% FCS, then plated at a concentration of 5×10^4^ cells per well and allowed to spread for 3.5 h at 37 °C and 5% CO_2_ [42,44]. Cells were washed three times in PBS, fixed with methanol, stained with Giemsa, and mounted in Kaiser gelatin (Merck). To quantify cell spreading, we photographed four separate fields using phase contrast microscopy. The spread cells showed a clearly defined halo of cytoplasm around the nucleus and were counted. Each experiment was performed in duplicate wells and was repeated at least three times.

### Statistical analysis

Statistical analysis was performed using SPSS 14 software (SPSS Inc., Chicago, IL). The Student-*t*-test for independent samples with a confidence interval of 95% was used to calculate statistically significant differences between samples. A p≤0.05 was considered statistically significant.

## Results

### Galectin-1 alters RPE cell morphology and binds the cell surface

**Figure 1:**
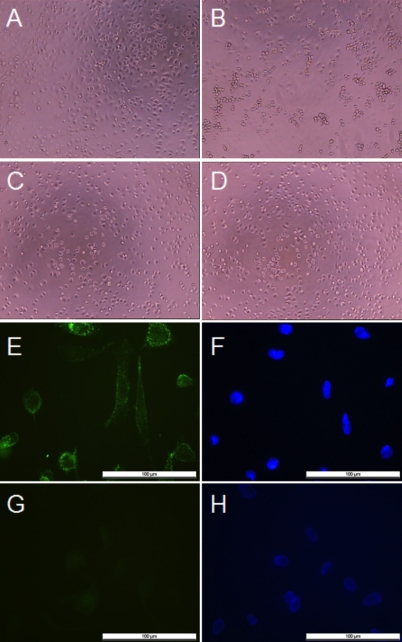
Effects of galectin-1 on the morphology of cultured human retinal pigment epithelial cells and cell surface binding of galectin-1. **A-D**: Human retinal pigment epithelial (RPE) cell suspensions were preincubated for 35 min without galectin-1 (**A**), or with 125 μg/ml galectin-1 (**B**), or 100 mM β-lactose before addition of 125 µg/ml galectin-1 (**C**), or 100 mM β-lactose (**D**) in the medium. RPE cells were then plated at a density of 0.5×10^4^ cells per well in 96-well plates and allowed to adhere for 90 min (**A**-**D**). The cells were observed by light microscopy (magnification 100×). **E-H**: Galectin-1 is detected on the surface of human RPE cells by immunofluorescence. Cells were cultured on glass coverslips for 16 h before being treated with biotinylated galectin-1 (**E**). They were fixed, then stained with a fluorescent streptavidin conjugate. For controls (**G**), untreated cells were exposed to streptavidin conjugate alone. Nuclei were counterstained with 1 µg/ml Hoechst 33342 (**F**, **H**). Localization of bound galectin-1 was visualized by fluorescence microscopy at a 400x magnification. The bar represents 100 μm.

To determine whether galectin-1 is capable of triggering cellular responses, we added purified protein to RPE cell suspensions before plating them. Cells were observed by phase-contrast microscopy. Regular cell spreading with intercellular spaces was invariably seen in untreated controls 90 min after plating (Figure 1A). In the galectin-1-treated cells, however, both the morphology and the cell density were markedly different (Figure 1B). Cell spreading was less frequent, with a predominance of rounded cells or loose cell clusters, which suggested that adhesion between cells was stronger than adhesion between cells and the substratum. This may in part arise from the cross-linking activity of the homodimeric lectin, which–when able to bind cells–has the capacity to agglutinate cells. To pinpoint carbohydrate-dependent binding as relevant, we included the inhibitor, β-lactose, in the assay panel. Its presence in the medium had no effect on cell morphology (Figure 1D). However, when galectin-1 was added in the presence of β-lactose to block its cell binding, agglutination and rounding of cells was restored (Figure 1C).

As a next step, biotinylated galectin-1 was added to assess the binding pattern of galectin-1 at the level of the individual cell. Immunocytochemical monitoring of cultured human RPE cells clearly revealed pronounced staining of the cell membranes, suggesting cell-surface binding of the lectin (Figure 1E-H). To exclude cytotoxic effects of this binding, MTT assays were performed to confirm cell viability.

### Lack of in vitro toxicity of RPE-bound galectin-1

**Figure 2:**
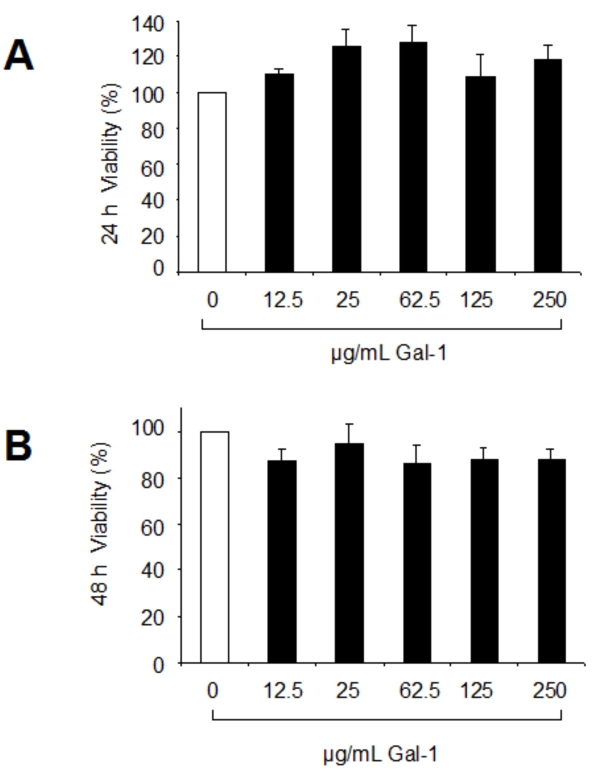
RPE cell viability was unaffected by galectin-1. Human RPE cells were seeded in 96-well plates at an initial density of 1×10^4^ cells/well in DMEM supplemented with 10% FCS. Cells were cultured overnight before the indicated concentrations of galectin-1 were added to the medium in DMEM containing 2% FCS, and cell survival was assessed by MTT assay after 24 h (**A**) and 48 h (**B**) of culture. RPE cells cultured with incubation medium alone served as controls. The values were normalized to untreated controls (100%) and represent the mean±SD of six experiments performed in duplicate wells.

In cells treated with galectin-1 for 24 h, MTT conversion rates reached 110% to 127% of the untreated control, indicating that survival was not affected by the presence of galectin-1 (Figure 2A). After 48 h exposure to galectin-1, the MTT conversion rate by RPE cells decreased by about 15% (Figure 2B). The observed reduction in cell number after 48 h may most notably be due to an antiproliferative effect of galectin-1, as indicated by [methyl-^3^H]thymidine uptake (data not shown).

### Effect of galectin-1 on RPE cell adhesion

**Figure 3:**
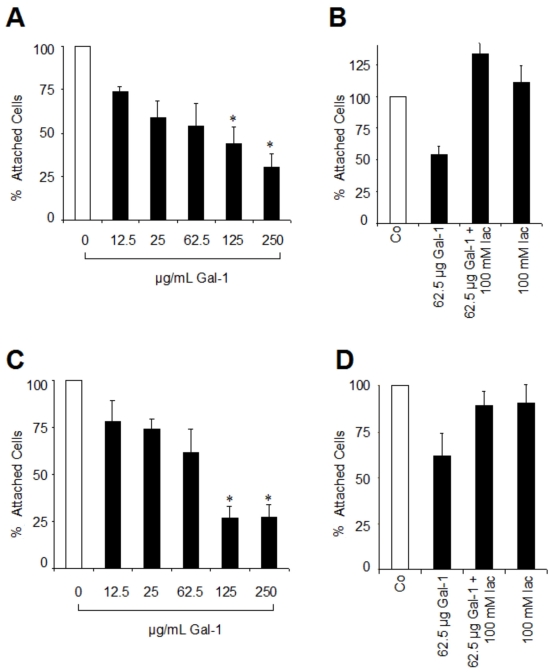
Effect of galectin-1 on RPE cell adhesion. **A**, **C**: Galectin-1 inhibits RPE cell attachment to Fn (**A**) and Lam (**C**) in a dose-dependent manner. RPE suspensions were incubated for 35 min with the indicated concentrations of galectin-1 (Gal-1) and then plated on wells coated with Fn or Lam at a density of 2×10^4^ cells per well. Values indicate means±SD of four experiments performed in triplicate and are expressed as percentage of controls without galectin-1 in the medium. **B**, **D**: The inhibitory effect of galectin-1 on attachment of RPE cells to Fn (**B**) and Lam (**D**) is mediated via carbohydrate binding. RPE cells were initially incubated in the presence or absence of 100 mM β-lactose (lac) as indicated. Values represent means±SD of three experiments performed in triplicate and are expressed as percentage of controls (Co) without galectin-1 or β-lactose in the medium. Statistical analysis was performed using Students *t*-test and a p value <0.05 was considered as statistically significant (*).

To test whether galectin-1 has an influence on RPE cell attachment to the ECM glycoproteins, Fn and Lam, we performed quantitative adhesion assays with untreated cells and with RPE cells preincubated with different concentrations of galectin-1 before they were plated. Galectin-1 pretreatment significantly decreased attachment to both glycoproteins in a dose-dependent manner. Pretreatment of RPE cells with 12.5 µg/ml galectin-1 resulted in a 26.4% (±2.9 SD) reduction in attachment to Fn, whereas 250 µg/ml galectin-1 inhibited attachment of more than 70% of treated RPE cells (Figure 3A). To block galectin-1 binding to the cells via its carbohydrate recognition domain, we performed control experiments in the presence of 100 mM β-lactose. The presence of β-lactose abolished the inhibition of RPE attachment caused by galectin-1. Clearly, attachment was dependent on carbohydrate binding (Figure 3B).

Comparable effects of galectin-1 on RPE cell attachment were observed for Lam-coated wells, where 72.8% (±11.4 SD; p<0.05) of RPE cells were unable to attach following initial incubation with 250 µg/ml galectin-1 (Figure 3C). As observed for attachment on Fn, this effect was completely eliminated in the presence of β-lactose (Figure 3D). These findings indicate that the inhibitory effect of galectin-1 on RPE attachment to both matrix glycoproteins is mediated via its lectin site.

To determine whether binding of galectin-1 to Fn is involved in the observed effect, we initially incubated 150 µg/ml soluble Fn with galectin-1 for 30 min before adding it to suspended RPE cells. In parallel experiments, RPE cells were initially incubated with soluble Fn alone, followed by incubation with 125 µg/ml galectin-1. Alternatively, cells were either left untreated (control) or treated with soluble Fn alone or with 125 µg/ml galectin-1 alone. Saturation of RPE cells with Fn alone had no effect on RPE attachment when compared to untreated control cells. However, preincubation with soluble Fn, followed by addition of galectin-1, or addition of Fn that had been initially incubated with galectin-1 resulted in an inhibitory effect on RPE attachment that was equivalent to that induced by galectin-1 alone (data not shown). The inhibitory effect of galectin-1 on RPE attachment may most likely arise from cell surface-binding of galectin-1, rather than from binding to or saturating Fn. This possibility would be in agreement with the immunocytochemical data. Furthermore, the presence of an excess of Fn or binding of galectin-1 to Fn does not interfere with the inhibitory effects of galectin-1.

**Figure 4:**
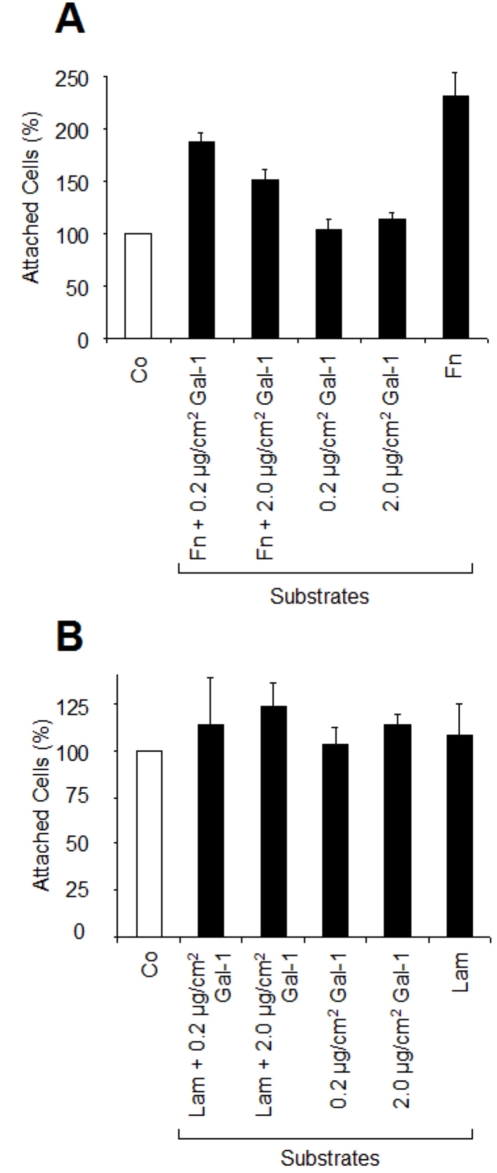
Galectin-1 as substratum does not support RPE cell attachment. RPE cells were seeded on 96-well plates precoated with Fn, or Fn+ Gal-1 (**A**), or Lam, or Lam+Gal-1 (**B**), or Gal-1 alone (**A**, **B**; see Methods section). Controls (Co) were plated on protein-free plastic. Values represent means±SD of three experiments performed in triplicate and are expressed as percentage of controls plated on uncoated plastic.

As part of delineating whether galectin-1 itself is a substratum for RPE cell attachment, we coated 96-well plates with 2 µg/cm^2^ of either galectin-1, Fn, or Lam, or a mixture of galectin-1 and glycoprotein. The overall attachment rate of RPE cells was higher on Fn- than on Lam-coated wells (Figure 4). In terms of numbers of attached cells per well, galectin-1, Lam, and uncoated plastic were equally attractive for RPE cells, suggesting that Lam does not promote, but also does not inhibit, attachment of RPE cells to tissue culture plastic. Fn markedly increased RPE cell adhesion to levels 231% higher than in the control uncoated wells. This increase was reduced by 44% and 80% when the Fn-coated wells had additionally been precoated with 0.2 µg/cm^2^ and 2 µg/cm^2^ galectin-1, respectively (Figure 4A). The combination of Lam with galectin-1 had no further influence on RPE attachment, which was equal to uncoated wells. These findings indicate that galectin-1 is not an adhesion substrate for RPE cells, and that it can even reduce the adhesive properties of Fn.

### Effect of galectin-1 on RPE cell migration

**Figure 5:**
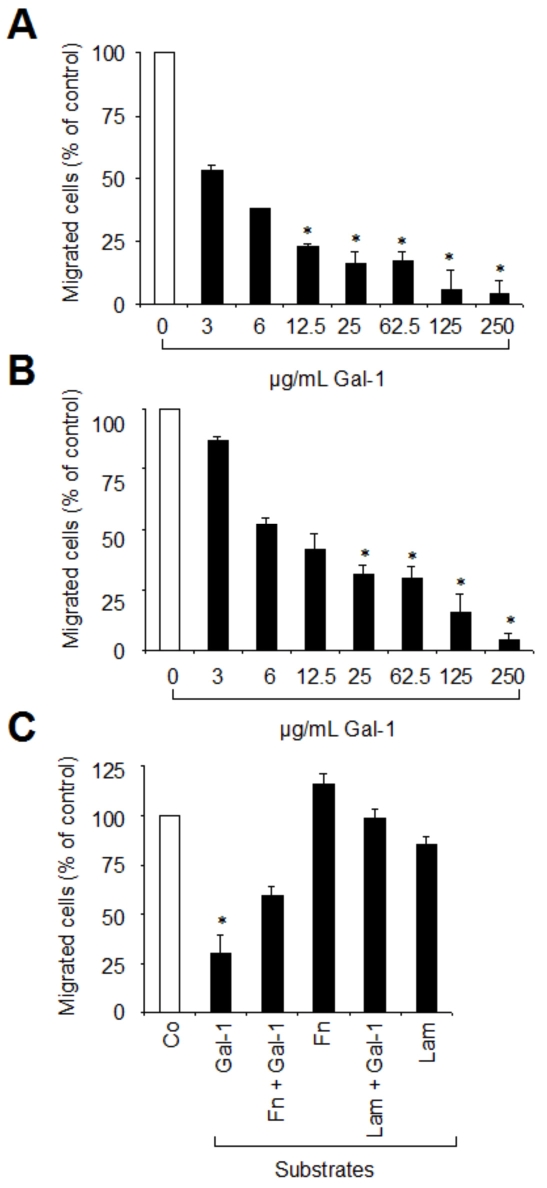
Soluble galectin-1 reduces RPE cell migration on fibronectin and laminin in a dose-dependent manner. **A, B**: Boyden chamber membranes were coated with either fibronectin (Fn; **A**) or laminin (Lam; **B**). RPE suspensions were incubated for 35 min with the indicated concentrations of galectin-1 and then 5×10^4^ cells were placed into the upper chamber. After five hours at 37 °C, cells that migrated into the lower surface of the filter were fixed, stained and quantified. Values indicate means±SD of four experiments performed in duplicate and are expressed as percentage of controls without galectin-1 in the medium. **C**: Galectin-1 as a substratum does not support RPE cell migration. Boyden chamber membranes were coated with either Fn alone, Fn+galectin-1 (Gal-1), Lam alone, Lam+Gal-1, or Gal-1 alone. RPE cells were then placed into the upper chamber at a density of 5×10^4^ cells per chamber and allowed to migrate as described above. Values indicate means±SD of three experiments performed in duplicate and are expressed as percentage of controls (Co). Statistical analysis was performed using Students *t*-test and a p value <0.05 was considered as statistically significant (*). RPE cells migrating on uncoated filters served as controls (Co).

To analyze the effect of added galectin-1 on RPE cell migration, we coated Boyden chamber membranes with either Fn or Lam. RPE cell suspensions were incubated in the presence or absence of different concentrations of galectin-1 before being placed into the upper chamber. To ensure an efficient directional migration, we added PDGF-BB to the lower chamber as a chemotactic agent. Pretreatment of RPE cells with different concentrations of galectin-1 caused a dose-dependent decrease of RPE cell migration in both cases (Figure 5A,B). At the highest concentration studied, galectin-1 inhibited migration by more than 90%.

Next, we studied the effect of galectin-1 as a substratum on PDGF-stimulated RPE cell migration. For this purpose, Boyden chamber membranes were precoated with 2 µg/cm^2^ of glycoprotein or galectin-1 or mixtures thereof. Overall, RPE migration on Fn and Lam was equal to migration on uncoated filters (Figure 5C), being slightly increased on Fn (116.0% ±5.6 SD) and slightly decreased on Lam (85.6%±3.8 SD). On membranes coated with galectin-1 alone, migration of RPE cells was reduced to 30.2% (±9.1 SD; p<0.05) of that seen for uncoated controls. To delineate whether saturation with galectin-1 affected RPE migration on Fn or Lam, membranes precoated with Fn or Lam were treated with galectin-1. Migration was reduced by a further 57% for Fn (Figure 5C). No inhibitory effect was seen for Lam. These observations correspond well with the findings for RPE cell attachment, inferring that galectin-1 does not stimulate RPE migration and can even attenuate migration on Fn. A hallmark of RPE in PVR is their extensive spreading, which prompted study of galectin-1 in this respect.

### Effect of galectin-1 on RPE cell spreading and morphology

**Figure 6:**
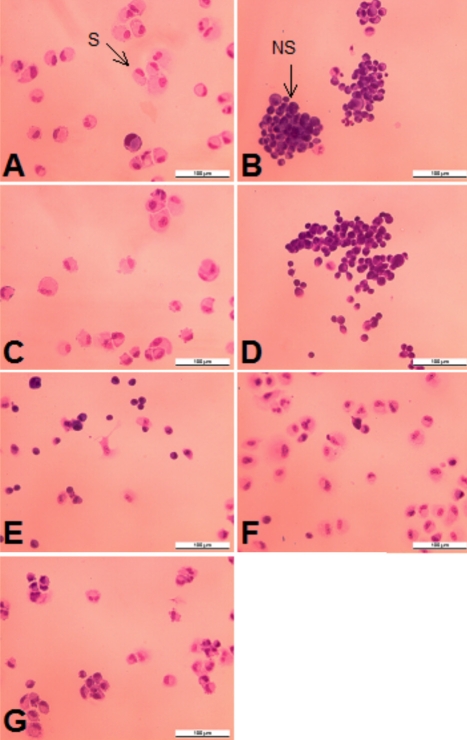
Morphology of cells used for collection of data in Figure 7. Representative light microscopic fields are shown. (**A**) Untreated RPE cells grown on Fn; (**B**) after preincubation with 250 μg/ml Gal-1 on Fn; (**C**) Untreated RPE cells grown on Lam; (**D**) after preincubation with 250 ug/ml Gal-1 on Lam; (**E**) Untreated RPE cells grown on Gal-1; (**F**) on Gal-1 and Fn; (**G**) on Gal-1 and Lam. Spreading cells (S) were defined as cells with cytoplasmic protrusions and perinuclear halo formation, and non spreading cells (NS) as rounded cells with little cytoplasmic spreading. The photographs represent a 100 fold magnification.

**Figure 7:**
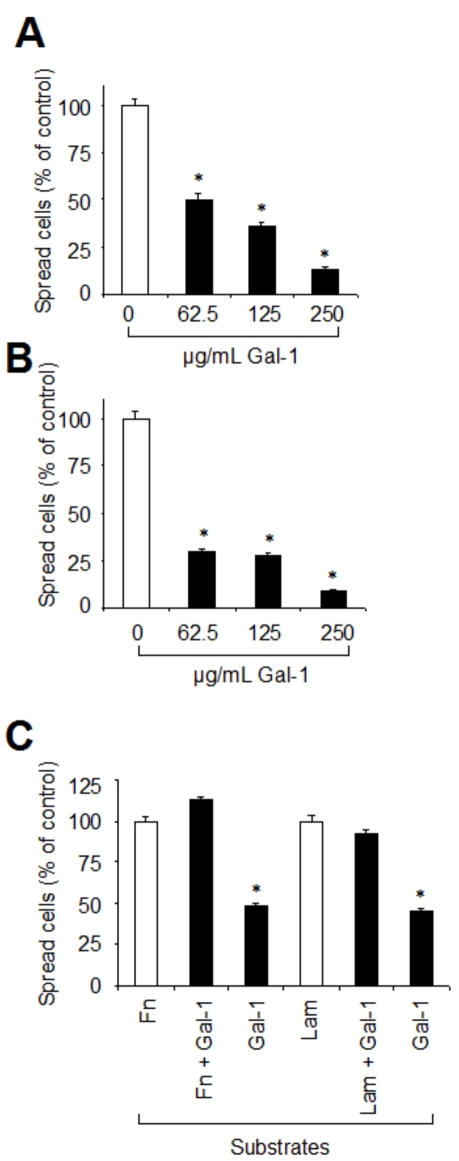
Galectin-1 inhibits spreading of RPE cells on fibronectin and laminin. **A, B**: RPE suspensions were incubated for 35 min with the indicated concentrations of galectin-1 and then plated on four chamber slides coated with either Fn (**A**) or Lam (**B**) at a density of 5×10^4^ cells per chamber. Cells were allowed to spread in DMEM for 3.5 h at 37 °C before fixation and Giemsa staining. Values indicate means±SD of four experiments performed in duplicate and are expressed as percentage of controls without galectin-1 in the medium. (**C**) RPE cell spreading on galectin-1 as a substrate. Four chamber slides were coated with either Fn alone, Fn+galectin-1 (Gal-1), Lam alone, Lam+Gal-1, or Gal-1 alone. RPE cells were then plated at a density of 5×10^4^ cells per chamber and allowed to spread. Results were obtained from evaluation of four separate fields by examining at least 100 cells per field. Values indicate means±SD of three experiments performed in duplicate and are expressed as percentage of cells spreading on Fn or Lam as a substrate. Statistical analysis was performed using Students *t*-test and a p value <0.05 was considered as statistically significant (*).

The influence of added galectin-1 on spreading of RPE cells was measured on four chamber slides coated with either Fn or Lam. RPE cell suspensions were pretreated with different concentrations of galectin-1 before plating. After 3.5 h of incubation, regular cell spreading with establishment of cytoplasmic protrusions, perinuclear halos, and intercellular spaces was invariably seen in untreated controls (Figure 6A,C). In the galectin-1-treated cells, however, both morphology and density of the cells were drastically changed. At all concentrations studied, cell spreading was less frequent, being replaced by a predominance of rounded cells lacking cytoplasmic protrusions or by loose cell clusters (Figure 6B,D). Quantitative assessment revealed an inhibition of RPE cell spreading in 86.9% (±1.4 SD; p<0.05) of the cells on Fn and 90.9% (±1.2 SD; p<0.05) on Lam, when cells had been incubated with 250 µg/ml galectin-1 before plating (Figure 7).

Finally, to analyze whether galectin-1 as a substratum had an impact on RPE cell spreading, four chamber slides were coated with the same substrates as described for migration and attachment experiments. When compared to Fn and Lam, spreading on galectin-1 alone was severely impaired, with about 50% of cells showing a lack of cytoplasmic protrusions and halo formation (Figure 6E, Figure 7C,). In combination with Fn and Lam, galectin-1 had essentially no inhibitory effect on RPE spreading (Figure 7C) and failed to modify cell morphology (Figure 6F,G).

## Discussion

Attachment of cells to the retinal surface, spreading along the surface, and migration with subsequent formation of cellular membranes [7,42] are the initial events in the cellular cascade that leads to PVR. In this study, we found that galectin-1 added to the medium of cultured RPE cells is an effective inhibitor of many of the processes involved in these early events of PVR pathogenesis. With no visible toxicity or effects on RPE viability, the lectin inhibited RPE attachment, spreading, and migration at concentrations less than 62.5 µg/ml. The galectin-1-mediated effects were dependent on the binding of β-galactosides, as ascertained by haptenic inhibition by β-lactose. As a substratum, galectin-1 impaired spreading and migration of RPE cells and profoundly reduced attachment to and migration on Fn. As previously demonstrated [45], we confirmed a clear substratum preference of RPE cells to Fn over Lam. Since Fn is a main component of the provisional ECM in early PVR, galectin-1-mediated inhibition of RPE attachment and migration on Fn, both when added exogenously and as a component of the ECM, underscores the potential of galectin-1 to modify the behavior of RPE cells on this important substratum.

When localized extracellularly, galectins are active in modulating cell-cell and cell-matrix interactions in a positive or negative manner, depending on the biologic and physiologic context, as has been shown experimentally for various types of human tumor cells [30]. Relevant bridging ligands include the poly-N-acetyllactosamine chains present on Lam, Fn, or integrins and branched glycans in glycoproteins or glycolipids [22,46,47]. In the present study, galectin-1 added to the medium in the dose range studied caused a marked reduction in adhesion, spreading, and migration levels of cultured RPE cells, accompanied by rounding and agglutination of the cells.

In view of the cell-surface binding of galectin-1, as determined by biotinylated galectin-1, it is possible that this lectin can exert its inhibitory activities on RPE behavior by blocking access of physiologic ligands to cell surface receptors. This would have the effect of disrupting focal adhesions, which could then result in the observed morphological alterations. For example, in carcinoma cells, relevant glycoprotein ligands of galectin-1 were identified as lamp-1 and lamp-2, carcinoembryonic antigen, and the fibronectin receptor [33,48-50]. In skeletal myocytes and human smooth muscle cells, galectin-1 interacts with α7β1 and α1β1 integrins, respectively [24,51]. Of note, β1-integrin plays an important role in RPE adhesion and migration on Fn and Lam. It has been shown in other cell types that galectin-1 modulates adhesive properties of cells by interacting with β1 integrins [24], thus modifying their ability to adhere to components of the ECM such as Lam and Fn [24,52,53].

Although the nature of the galectin-1 counter-receptors on the RPE cell surface is not yet known, it is likely that galectin-1 might be involved in the modulation of cell-matrix interactions. The result would be a decrease in the adhesive and motile properties of dedifferentiated RPE cells via interaction with functional glycans on the cell surface. This notion is further underscored by the finding that RPE cell behavior was sensitive to the presence or absence of galectin-1 on the culture substratum. When exposed to surface-presented Fn and galectin-1, the level of attachment and migration was markedly reduced when compared to Fn as a substrate alone. This again raises the possibility that galectin-1 may compete with Fn for ligands, or may block its access on the cell surface. In contrast, none of the activities tested was sensitive to galectin-1 as a substratum bound to Lam, most likely because Lam, per se, reportedly has only weak affinity for RPE cells [42,45].

As noted previously, a salient aspect of galectin-1 activity is its specificity in a given cell type. In agreement with our findings, galectin-1 can decrease migration in different cell types including colon cancer cells [54], smooth muscle cells, and eosinophils [24,55]. In contrast, in astrocytic tumors, galectin-1 increases cell migration [56], and has recently been reported to increase adhesion, proliferation and migration, in combination with vascular endothelial growth factor, in human umbilical vein endothelial cells, as a result of neuropilin-1 binding [57]. Comparable cell type-dependent functional divergence has been reported for cell attachment: galectin-1 can stimulate tumor cell attachment to Lam and Fn [26,30,31], but adhesion and spreading of skeletal myoblasts and vascular smooth muscle cells on Lam is inhibited [24]. Clearly, it will now be essential to provide insights into the molecular and regulatory mechanisms involved in the inhibitory effects of galectin-1 on RPE behavior.

In their review, Gabius et al. [58] noted galectins may interact with various types of (glyco)protein, depending on their cellular location. In a previous study, we found reduced RPE migration on Lam after silencing galectin-1 expression by siRNA transfection [37]. At first sight, these results may appear conflicting. However, reduced overall galectin-1 transcription will likely have ramifications on gene expression, which could account for the observed effect. Stable inhibition of galectin-1 expression in a glioblastoma cell line diminished the expression of several genes that either directly or indirectly influence adhesion and motility [59]. Only recently, Kleinman et al. [60] revealed that, irrespective of its target, siRNA binds to cell-surface toll-like receptor-3 (TLR-3) on RPE cells and triggers TLR-3 signaling, thus altering RPE function independent of any RNA-interference effect. This raises the possibility that the TLR-3-mediated siRNA-class effect may have accounted for the observed reduced migratory activity following galectin-1 siRNA transfection. However, this contribution may have been only partial, since scrambled siRNA did not significantly reduce RPE migration.

In summary, our results indicate that galectin-1 is able to inhibit early cellular events in PVR pathogenesis–namely, attachment, migration, and spreading of RPE cells–in a dose-dependent manner and at nontoxic concentrations. When looking at lectins, it should be noted that working with an endogenous protein will likely exclude the immunogenicity encountered with plant proteins, such as the mushroom lectin aforementioned. At any rate, it is wise to view these in vitro data with adequate caution. During PVR or vitreoretinal surgery [61], serum (glyco)proteins containing a considerable number of potential glycan ligands may attenuate the inhibitory effect of added galectin-1 on RPE cell behavior; thus, increased lectin concentrations may be required. In addition, a panel of ECM proteins beyond Fn and Lam has been found in PVR but has not yet been studied. Furthermore, ligands for galectin-1 are present in the interphotoreceptor matrix [62,63] or on endothelial cells [57,64]. Therefore, for a future in vivo application, possible deleterious effects of intravitreally administered galectin-1 on neighboring tissues certainly will have to be excluded.

Nevertheless, we have shown that an endogenous lectin, produced by human RPE cells, can profoundly and differentially affect the behavior of RPE cells in contact with major ECM components present in PVR. Given the combined effect of galectin-1 on RPE attachment, migration, and spreading, and its dose-dependent titratability on RPE behavior, this lectin deserves further investigation as a possible novel agent for the management of PVR or other anomalous wound-healing disorders.
